# Enhancement of the geological mapping in weathered plutonic and metamorphic rocks areas using village water supply wells data (lithology and hydrochemistry)

**DOI:** 10.1016/j.heliyon.2024.e40376

**Published:** 2024-11-14

**Authors:** Cheik Abba Cissé Ouangaré, Séta Naba, Patrick Lachassagne

**Affiliations:** a Laboratoire Géoscience et Environnement, (LaGE), Département des Sciences de La Terre, Université Joseph Ki-Zerbo, Ouagadougou, Burkina Faso; b HSM, Univ. Montpellier, CNRS, IRD, IMT Mines Alès, Montpellier, France

**Keywords:** Geological mapping, Granitoids, Metamorphic rocks, Silica, Village water supply boreholes

## Abstract

In weathered plutonic and metamorphic rocks areas, because rock outcrops are rare due to the extensive regolith cover, geological mapping is largely based on the interpretation of airborne data and imagery (aerial photographs, satellite images, airborne geophysics when available, etc.). In the sub-Saharan Africa, numerous village water supply campaigns were performed during the last 40 years. Most hydrogeological and geological data from these campaigns are stored and safeguard in country scale databases. In this study, we develop a methodology to improve the existing geological maps in a such geological context with lithological data from the existing databases. These data were not used during past geological mapping; yet they provide a complementary access to the lithology, notably in areas where outcrops are very scarce. We propose a coherent methodology to use and validate such geological data, complementary to field observations on the scarce outcrops, to cope with their uncertainties. We also show that groundwater hydrochemistry, namely the silica content, can be of use to complete the geological mapping. Using data from 735 boreholes drilled in the Koudougou and Léo square study area of Burkina Faso, we validate this new methodological approach and locally propose a more accurate 1:200,000 geological map.

## Highlights

• We propose a new cartographic approach in basement zones with thick regolith cover, based on data from lithological descriptions of village hydraulics water boreholes.
• We demonstrate the value of hydrochemistry, in particular the silica content of borehole water, for discriminating major lithological units in basement zones.
• We propose a methodology to check and validate the boreholes' lithology and, endly and if necessary, to correct the existing geological maps.

## 1 Introduction

A geological map is a representation on a topographic base, of the geological formations that outcrop or are hidden (by soils, vegetation, buildings …) within a specific area [1]. It is a essential tool for the geologist, facilitating the understanding of spatial relationships between geological formations and their environment context [2,3]. It enables mining, hydrogeological and energy resources, etc. to be visualised and characterised more accurately at a single glance [4] and provides a solid basis for land-use planning. All these possibilities are all the more relevant when the geological map is reliable, i.e. in line with the realities on the ground. Although necessarily an imperfect representation [5], geological maps are designed to represent this fidelity of the terrain, to the extent that they call on a diversity of sources, techniques and approaches from the Earth sciences [6]. Today, geomodelling is one of the approaches commonly used in geological mapping. It provides 3D geological models by means of various mapping software packages [5,7]. However, since classical geological maps, these models are an approximation of reality and can be erroneous, especially outside areas where field observations are dense.

In Burkina Faso, the geological maps produced by the 2003 SYSMIN project remain the most commonly used. In the Koudougou and Léo sheets or square degrees, as for many other sheets in Burkina Faso, the most detailed maps are at a scale of 1:200,000. At this scale, the density of exploration in the field is very low. In addition to geological mapping, indirect methods such as photo-interpretation, airborne geophysics, rock geochemistry, etc. are used [6,[8], [9], [10]]. As a result, the geological contours and features drawn up in this way do not always correspond to the reality on the ground, despite the care taken in drawing them up [8]. This is the case of the geological maps of the Koudougou and Léo sheets, produced under these conditions, and probably of the other basement zones, made up of the same crystalline and metamorphic rocks and covering more than 80 % of Burkina Faso territory. In these basement areas of sub-Saharan Africa, rock outcrops are very rare in the field due to the thick regolith cover and relatively flat topography. This seriously hampers lithostructural mapping in the field [10]. Furthermore, due in part to differential weathering [11,12] these outcrops may not be representative of the most represented lithologies.

In such conditions, borehole data (mining or hydrogeological) are the only additional means of direct access to non out cropping geological formations. Whereas mining borehole data are not widespread and may not be easily accessed, Water well drillers' records may be the largest source of data available to describe subsurface geological formations [8].

However, they have been used very little in the production of geological maps in Burkina, even though they can provide valuable information to improve the accuracy and reliability of geological maps, provided that the geological descriptions made during drilling are accurate and their interpretation consistent with the one of the geological map.

In Burkina Faso, as in most West African countries, the advent of the International Drinking Water Supply and Sanitation Decade in the early 1980s was a turning point for the drilling of thousands of boreholes to supply water to the population [15]. Of a forecast of 100,000 water points to be built by the end of the decade in the twelve African states concerned, 27,000 had already been completed by the end of the 1980s [16]. In 1986 alone, at least 1470 boreholes were drilled in West Africa's basement zones [17]. Most hydrogeological and geological data from these campaigns are stored and safeguard in country scale databases (see for instance Refs. [18,19]). In 2010, about 16000 water wells were available in hydrogeological databases only in Burkina Faso [19].

The aim of this paper is to determine whether the use of lithological data from water boreholes can help improve geological mapping in weathered basement areas. It also aims to highlight the added value of these data in combination with conventional geological mapping methods to improve geological maps in basement areas. Finally, this paper invites geological surveys to take these data into account during future mapping campaigns in the basement regions of Burkina Faso, and in other basement regions of the world where few outcrops are observed. In view of the diversity of terminologies that can be used by drillers and or hydrogeolologist to describe the rocks identified during drilling, we propose a methodology to validate this data by combining field observations with groundwater silica content analysis.To illustrate this approach, we utilize lithological data from 735 water boreholes drilled in the Sanguié province as a case study for the development of this new approach to geological mapping in the basement zone of Burkina Faso. The province of Sanguié, like the other provinces of Burkina Faso, has benefited from the drilling of around a thousand boreholes through various village water supply programmes. Two of the province's communes, Dassa and Kyon, where the above-mentioned conditions are met, have been used to test the new approach in the field. The new approach was validated by producing an updated geological map of the study area at the same scale as the reference maps, taking into account geological data from boreholes and/or field observations, and the silica content of groundwater.

Beyond the present introduction, which provides the justification and objectives of this study, the article is structured into three main sections: the Materials and Methods section, which presents the environmental context of the study area as well as the methodology developed; the second section outlines the Results of this methodology; finally, a Discussion analyzes these results and explores their implications. We conclude with a Conclusion that summarizes the contributions of this study and proposes perspectives for future research.

## 2 Materials and methods

### 2.1 Context of the study area

**Fig. 1:**
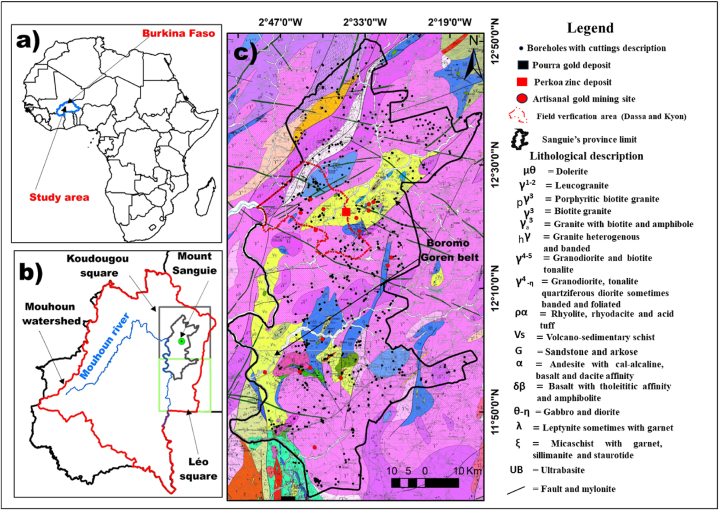
Context of the study area: a - Location of Burkina Faso in Africa; b- Location in the Mouhoun watershed and c- Geological map of the study area from the 1:200,000 geological map [13,14].

The province of Sanguié is one of the 45 provinces of Burkina Faso, a country situated in the heart of West Africa (Fig. 1a). Geographically, the central part of the province lies between latitude 11°30′ and 12°50′ N and longitude 2°10′ and 2°55′W (Fig. 1c).

The Sanguié province lies mainly on the Koudougou geological map sheet and a small part on the Léo map sheet (Fig. 1b). It is entirely included in the Mouhoun catchment area, which takes its name from the Mouhoun river, one of the most important perennial watercourses in the country (Fig. 1b). Like the rest of Burkina Faso, the province is characterised by a fairly flat and monotonous morphology, generally not exceeding 400 m in altitude [9]. Mount Sangyè, which lends its name to the province, is the highest point, culminating at an altitude of around 400 m amsl [20].

The subsurface geology of Sanguié consists of metaplutono-volcanic, metasedimentary and granitoid formations (Fig. 1c). The metaplutono-volcanic and metasedimentary formations, known as birimian formations [21] are grouped together in the greenstone belts. The regional metamorphism that affects them is of the greenschist facies and locally reaches the amphibolite facies around the major plutons. All these formations are Paleoproterozoic in age (2.5–1.8 Ga). Metamorphism, deformation and the peak of plutonic activity occurred around 2.2t o2 Ga during the Eburnian orogeny [22]. But it is also recognised that shortening and deformation affected the Birimian terrains diachronically in a SE to NW direction ([23] and references therein).

Burkina Faso has a number of greenstone belts, including the Boromo-Goren belt, one of the richest in gold mineralization and other metals such as Cu, Au, Co, Ag, Zn, Pb, etc. It crosses the province in a NW-SE direction, leading to gold panning activities in many villages in the province (Fig. 1c). It has also been the subject of numerous mining prospecting and scientific research projects [10,[24], [25], [26], [27], [28], [29], [30]].There are at least 15 artisanal gold mining sites in the province [31]. The Perkoa zinc deposit (Fig. 1c), located in the commune of Réo, is the only sulphide cluster-type base metal deposit recognised in Burkina Faso [9].

Hydrogeologically, the Sanguié province, like other regions of the West African basement, is characterised by the exploitation of groundwater through the saprolite captured by traditional large-diameter, shallow wells or through the underlying fractured layer captured by boreholes [18,19,32,33]. Wells are usually less than 15 m deep, while borehole rarely exceed 100 m. Groundwater levels are relatively close to the surface, facilitating vegetable cultivation and other forms of agriculture The local population is mainly involved in farming and livestock rearing, alongside gold panning, which is increasingly attracting younger people.

### 2.2 Methodology developped

The methodology developed in this research is based on lithological description data from water boreholes drilled in the Sanguié province during various village water supply projects available in existing data bases. During the drilling process, lithological description was obtained from the drilling cuttings interpreted by the driller or the hydrogeologist overseeing the operation. Consequently, these lithological descriptions are often less precise and accurate than those provided by geologists responsible for geological mapping. For this research, we compiled these lithological data into a newly created database (Table 1). To assess the quality and validity of these data, geological fieldwork was conducted and groundwater samples were collected for hydrochemical analysis, with focus on dissolved silica content. In addition to the lithological data from the boreholes, we used the geological maps of the study area, the most detailed of which are at a scale of 1:200,000 [13,14].

**Table 1:** Summary of primary borehole data from the database compilation.

Parameter	Methods	Unit	Reference	Total	Available information on boreholes (%)	Observation
Type of construction	Down-the-hole hammer	Borehole		819	100	Boreholes were drilled with down-the-hole hammer using drilling workshops set up during the various village hydraulics campaigns.
Geographic coordinates	GPS	Degrees Minutes Seconds (DMS)	WGS 84 Zone 30N	819	100	The coordinates of were recorded by GPS when the borehole was drilled. The coordinates were mainly recorded in sexagesimal system (DMS). For the purposes of this work, all coordinates have been converted to projected coordinate system (UTM).
Drilling period			Year of construction	819	100	The boreholes were drilled between 1982 and 2021 by several village water projects financed by the government and its development partners: Conseil de l'Entente (CE), Banque Islamique du Développement (BID), Banque Africaine de Développement (BAD), Appui Budgétaire Sectoriel (ABS).
Borehole lithology	Description of drilling cuttings			735	89.7	The cuttings are described in 1 m increments as the drilling progresses until drilling generally stops at the top of the sound rock.

The field work enabled us to make observations on 17 outcrops and to take 5 rock samples (3 at Dassa and 2 at Kyon) with a view to preparing thin slides for polarising microscope observations.

**Fig. 2:**
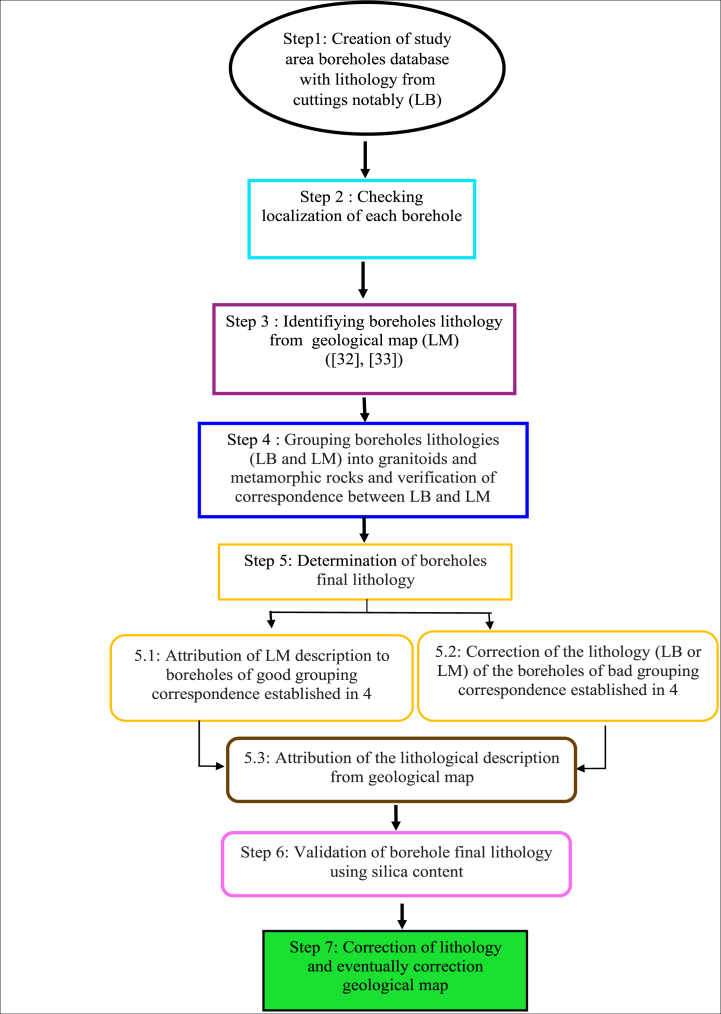
Simplified flow chart of the main stages of the developed methodology for correcting the geological map.

Fig. 2 summarizes the seven main steps followed in developing this new geological mapping approach. Finally,we propose an updated geological map of the Sanguié province; delineating the revised boundaries of geological formations.

#### 2.2.1 Creation of study area boreholes database (Step1)

We compiled the data from the water boreholes drilled in the frame of village water supply projects in the Centre Ouest region of Burkina Faso with a view to compiling a borehole database. Most of the data are available in technical reports in paper format, with only a small portion in digital format, stored in the archives of the Regional Directorate in charge of Water and Sanitation in the Centre Ouest region, or from private structures involve in these projects such as BRGM, ANTEA, and others. We obtain access to multiple technical reports covering the period from 1982 to 2021 [34,35].

To compile the database, all the available data were digitized. The main parameters taken into account in the database are (Table 1):

- type of drilling: large-diameter well or borehole;
- location: well/borehole drilled in the Sanguié province or elsewhere;
- the period (year) when the borehole was drilled;
- the identification of the project for which the borehole was drilled;
- the borehole lithology (LB) described from cutting collect and interpreted during drilling by the driller or an hydrogeologist.

#### 2.2.2 Checking the geographical coordinates associated with each borehole in the database (Step 2)

Given that some of the data dates from before the widespread use of satellite positioning systems, it was necessary to verify the geolocation of the boreholes included in our study. This verification of borehole location was conducted at the provincial, commune and village level respectively. The village is the smallest administrative unit in Burkina Faso [20].

#### 2.2.3 Identifying the lithology of each borehole from the geological map LM (Step 3)

For this identification, we used the 1:200,000 geological map of the study area [13,14].

#### 2.2.4 Lithology grouping and verification of the correspondence between the two lithological data sets (Step 4)

Several lithologies were described during borehole drilling (LB) and geological mapping (LM) as shown in Table 2. This table compares between the lithology identified on the geological map (LM) with the lithological description made during drilling, from cuttings (LB).

**Table 2:** Identification on the geological map of the lithology (LM) associated with each borehole described from the cuttings (LB) and their grouping into granitoids and metamorphic rocks. The table can be read as follows: 476 boreholes identify from borehole cuttings at drilling as granites, are identified on the geological map as corresponding to 258 biotite granite, 74 porphyritic biotite granite, …,2 Andesite with calc-alkaline, basalt, and dacite affinity.

Lithology				Geological map rocks grouping										
				Granitoids					Metamorphic rocks					
				Lithology identified from geological map (LM): N										
				Biotite granite: 379	Porphyritic biotite granite: 114	Granite heterogeneous and banded: 35	Leucogranite: 36	Granodiorite, tonalite, and quartziferous diorite sometimes banded and foliated: 55	Volcanosedimentary schist:41	Basalt with tholeitic affinity and amphibolite: 41	Gabbro and diorite: 12	Leptynite sometimes with garnet: 11	Rhyolite, rhyodacite, acid tuff: 8	Andesite with calc-alkaline, basalt, and dacite affinity: 3
**Boreholes lithological description rocks grouping**	**Granitoids**	**Lithology identified from borehole cuttings (LB): N**	Granite: 476	258	74	17	27	43	16	21	5	7	6	2
			Biotite granite: 5					1	2		1			1
			Pink granite: 39	28	7	1				1	1		1	
			Amphibole-biotite granite: 2	1						1				
			Amphibole granite: 5	2	1					1	1			
			Grey granite: 4	1	3									
			Melanocrat granite: 1			1								
			Dark granite: 1										1	
			Pegmatite: 28	20	1		4	2	1					
			Granitoids: 6	3					2	1				
			Granodiorite: 8	3		1		3		1				
			Diorite: 22	10	4	3	2		1	2				
			Microgranular arena: 1	1										
			Granitic arena: 2	2										
			Quartz veins: 13	2	5	1	1		3			1		
			Sand: 7	5	2									
			2 Syenite	1	1									
			Lateritic clay alteration: 1				1							
	**Metamorphic rocks**		Metamorphosed granite: 5						2	2	1			
			Black metamorphic granite: 3							2	1			
			Migmatite or Granito-Gneiss: 7	3	1	1		1		1				
			Gneiss: 9		3	4		1	1					
			Migmatite: 10	3	2	3				2				
			Amphibolite: 8	1	3	2		1	1					
			Micaschist: 3	3										
			Schale: 21	11	2		1	1	2	2	1	1		
			Metamorphic schist: 27	13	2	1		1	6	2	1	1		
			Green Rock: 5	2					2			1		
			Greenstone or Volcano-sedimentary rocks: 9	4	2			1		2				
			Metavolcanic (Métabasalte): 2	1	1									
			Dolerite: 3	1					2					

To harmonise and incoporate all the descriptions, we grouped them in two main groups: the granitoid group, which includes lithologies from the geological map such as Tonalite - Trondhjemite -Granodiorite (TTG), and granites, and the metamorphic rocks group, which includes leptynites, metavolcanic rocks, basic to intermediate metaplutonic rocks and metasedimentary rocks (metarhyolites, metagabbros, metaandesites, metabasalts and metadiorites, metaarenites, sericite schists, etc.). We then examined the correspondence between the lithological grouping from both datasets (Table 3). This table shows the verification of the match between the lithological groupings from the borehole descriptions and those the geological map. At this stage, the grouping lithology described in the borehole is compared with the lithological grouping shown on the geological map.

**Table 3:** Verification of the lithological correspondence between the two lithological data sets (LB and LM grouping).

Lithology grouping	Lithology identified from borehole cuttings (LB)	Lithology identified from geological map (LM)	No description match	
			Number of boreholes	Rate (%)
Granitoids	623	619	80	10,9
Metamorphic rocks	112	116	76	10,3
Total	735		156	21,2

#### 2.2.5 Determination of the definitive lithology of the boreholes in the study area (Step 5)

**Fig. 3:**
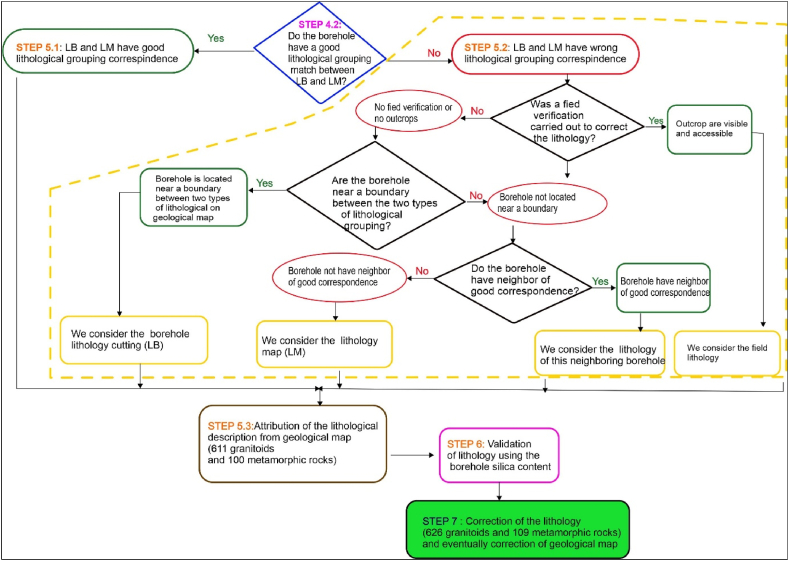
Flow chart for determining and validating water well lithology: LB = lithology determined from borehole cuttings; LM = lithology determined from the geological map [13,14].

The final lithology assigned to each borehole in the study area was validated after comparing the conformity between the two datasets as explained in section 2.2.4 above. Two cases can be observed, as shown in Fig. 3 below:

− 2.2.5.1 Conformity of the two lithological data sets (LM and LB)

When the two datasets are consistent, we are in the case where the lithological descriptions of the two sets of data can be considered to be identical. The final lithology is taken to be the name of the rock described on the geological map (LM).

− 2.2.5.2 No-conformity of the two lithological data sets

If the two datasets do not match, the discrepancy may be due either an incorrect or incomplete description and interpretation during borehole drilling or to inaccuracies in the geological map that need to be corrected.

In this case, a new lithology must be assigned to the borehole in question. We propose the following approach, that we named the "**geological mapping methodology using borehole lithological data**".

This methodology first relies on field verification as a criterion for correcting the lithology. For some boreholes, lithology was confirmed through macroscopic observations on outcrops and samples and thin sections. Where necessary, the lithology of the borehole is corrected. If it has not been possible to validate the lithology in the field (due to lack of outcrops or no field observations), we propose that the lithology be validated using data from neighbouring boreholes with a good lithological description established in step 4 (drilled in the same district or village). The following two conditions must be met:

##### 2.2.5.1 Borehole located near a boundary between two lithologies on the geological map

This situation corresponds to the case where it is the accuracy of the map that may be responsible for the poor match. In this case, we consider the lithology of the description based on the cuttings (LB).

##### 2.2.5.2 Borehole not located near a boundary between two lithologies on the geological map

This situation corresponds to the case where the lithology identified from the borehole (LB) was not indicated on the map. In this case, the following two criteria should be considered when correcting the lithology:

i) The borehole is located near one or more neighbouring boreholes drilled in the village or district where a good LB and LM match was established in step 4. Then, the lithology of the neighbouring borehole or boreholes (LB) is considered;
ii) The borehole has no neighbours, so we considered the lithology of the geological map (LM).

#### 2.2.6 Validation using groundwater hydrochemistry

The hydrochemical approach involved, during this research project, sampling boreholes on the field and analysing several physico-chemical parameters, including temperature, pH, electrical conductivity, major and minor elements and dissolved silica in groundwater in the communes of Dassa and Kyon in the Sanguié province. The objective was to identify chemical distinction according to the type of aquifer tapped by each borehole. Among the many parameters analysed, only silica (SiO_2_) was provedrelevant distinguishing between waters based on lithology. The silica present in groundwater comes exclusively from water-rock interactions [36]. It is considered as a good tracer, making it possible to explain and discriminate between the lithology of major geological units [[37], [38], [39]]. Silica is also a reliable indicator of residence time in crystalline rock aquifers [40]. The silica content of groundwater sampled in the boreholes was used in combination with field observations to validate the final lithology derived from the drillers' description.

The silicon (Si) content of water samples, collected during both high and low piezometric levels was analysed using inductively coupled plasma mass spectrometry (ICP-MS), iCAP TQ (Thermo Scientific®) on the AETE-ISO platform (OSU OREME, University of Montpellier), with an analytical precision within a few percentage points. The accuracy of the measurement was verified by comparing the measured concentrations with compiled reference values [41] from the water reference material, SLRS6 (NRC Canada). The difference between the measured values and the compiled value was less than 5 %. ICP-MS provides exceptional precision in detecting nanoparticles and dissolved major elements [[42], [43], [44]]. The silica content (SiO2) was then calculated from the mass ratios between Si and SiO_2_. A total of 45 boreholes in the communes from Dassa and Kyon were sampled and analysed during both the dry season (October to December 2021) and the wet season (March to April) 2022 respectively.

#### 2.2.7 Correction of lithology and geological map

This final step allows for the correction of the borehole lithology based on the previous stages and, subsequently, the map by proposing new lithological boundaries.

## 3 Results

### 3.1 Application of the methodology to data from water boreholes in the Sanguié province

#### 3.1.1 Database of water boreholes in Sanguié

Table 1 presents a total of 819 boreholes compiled to create a database of boreholes for the Sanguié province as part of this research. All boreholes were drilled within Sanguié province. The geographical coordinates of each borehole were recorded using a GPS receiver at the time of drilling. 735 boreholes representing 89.7 %, have a lithological description based on the cuttings described during drilling. 84.2 % and 15.8 % of these descriptions were made respectively before and after the 2003 geological map was produced. Until 2003, the monitoring and control of the drilling work was mainly conducted by geologists from the Bureau de Recherche Géologique et Minières (BRGM) and its subsidiary Antea.

The boreholes were drilled in the province as part of several village water supply projects carried out between 1982 and 2021. The most notable projects in which boreholes have been drilled include the Council of the Entente (CE) project, carried out in three phases from 1983 to 1998, the two phases of the Islamic Development Bank (IsDB) project, carried out respectively between 1995 and 1998 and again from 2001 to 2003. Additionally the recent projects carried out by the African Development Bank (BAD) in 2015 and the Sectoral Budget Support(ABS) from 2010 to 2021.

#### 3.1.2 Verification of the geographical coordinates of the boreholes in the database

Verification of the data revealed that some of the boreholes were poorly georeferenced [19]. observed a similar issue in the Ouahigouya area, where the coordinates of water boreholes scattered around certain villages were often associated with the geographical coordinates of the village center. In the case of the Sanguié province, 200 boreholes in the database, drilled accross 61 villages, were linked to one, two or three points within the village wherethey were drilled. We can speculate that the fact that these points were attached to more than one point in the same village was certainly linked to the size of the village. Also, 4 boreholes were in the wrong geographical position in relation to their location on the map. It should also be noted that, although the geographical coordinates are identical for some boreholes in the same village, they are distinct with different technical data (borehole depth, saprolite thickness, final discharge). The lithologies described also varied for some of these water boreholes. Of these 204 boreholes (200 + 4), 168 boreholes showed a similar description between the lithology described for the borehole and that of the geological map. These boreholes are all located in areas where the geological map only mentions granitoids or metamorphic rocks. This shows that the imprecision of their location has no impact. 32 boreholes did not match the two sets of lithological data. These were validated using the same methodological approach applied in step 5. However, these 204 boreholes were not used to correct the geological map.

#### 3.1.3 Borehole lithologies identified from the geological map (LM)

The lithological description of 735 boreholes, based on cuttings (LB) and, employs 31 distinct terminologies a. The 1:200,000 geological map indicated, that these same boreholes are positioned on eleven (11) lithologies (Table 2). The lithologies listes in decreasing order of abundance, include, biotite granite (379), porphyritic biotite granite (114), granodiorite, tonalite and quartz diorite, sometimes banded and foliated (55), volcano-sedimentary schist (41), basalt with tholeitic affinity and amphibolite (41), leucogranite (36), heterogeneous banded granite (35), gabbro and diorite (12), leptynite sometimes with garnet (11), rhyolite, rhyodacite, acid tuff (8) and finally andesite with calc-alkaline affinity, basalt and dacite (3).

**Fig. 4:**
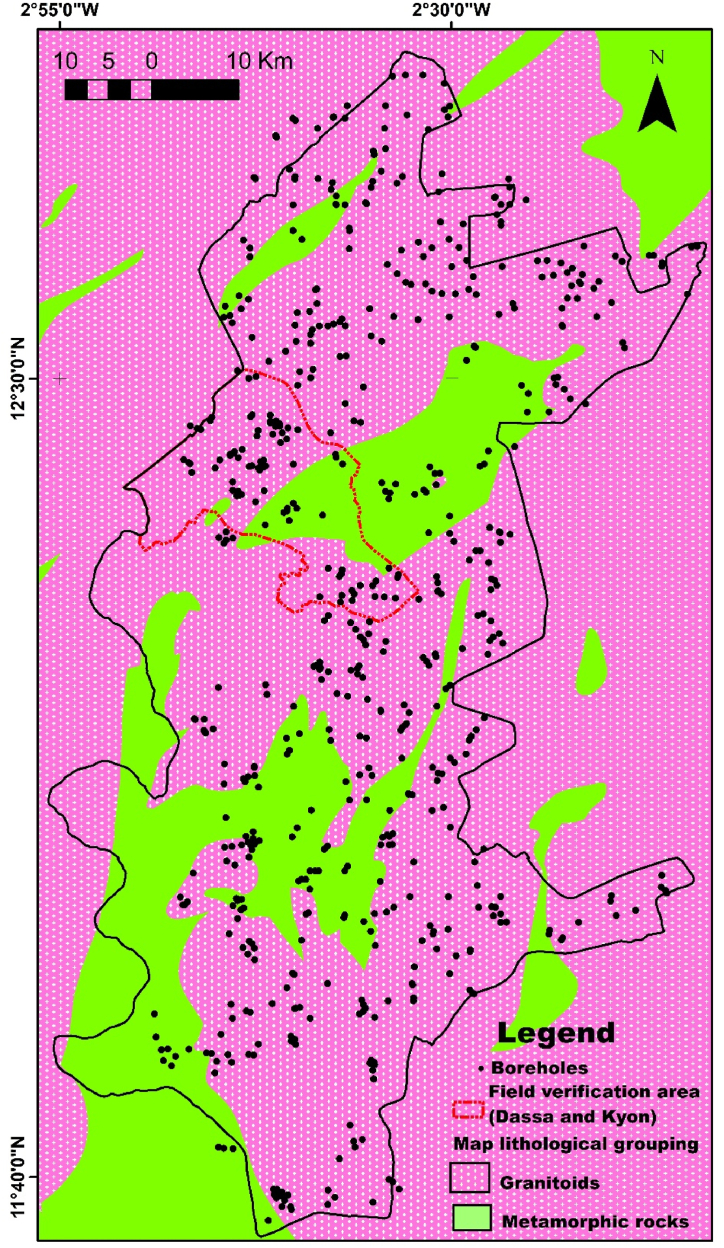
Grouping of lithologies in the study area into granitoids and metamorphic rocks and identification of associated boreholes. On the map, the granitoid group includes (Tonalite - Trondhjemite -Granodiorite (TTG), and granites) and the group of metamorphic rocks, the other lithologies than the granitoids (rhyolite, volcano-sedimentary schist, sandstone and arkose, basalt, leptynites, gabbro and diorite, mica schist, ultrabasite, etc.).

The lithologies derived from the cuttings, like those derived from the geological map, have been grouped into two major families of basement rocks: granitoids, comprising geological formations close to granite and Tonalite-Trondhjemite-Granodiorite (TTG), and metamorphic rocks comprising lithologies other than granites and TTG (Fig. 4). Regardless of the type of lithological dataset used, granitoids are more abundant than metamorphic rocks in the Sanguié province. Granitoids account for more than 80 % of the basement rocks in the study area, compared with just under 20 % for metamorphic rocks in each of the two lithological datasets.

#### 3.1.4 Comparison of the two lithological data sets

If we include all the lithologies described (LB) and identified (LM) respectively in the granitoid group on the one hand and in the metamorphic rock group on the other, we can see that in 21.2 % of cases, the lithological types at boreholes (LB) are the inverses of the types shown on the geological map (LM) at the same location. These discrepancies (Table 3) deserve to be reviewed and corrected. Of 65 water boreholes described in the cuttings as tapping volcano-sedimentary schists, 44 were identified on the geological map as corresponding to granitoids. The other lithologies in this group (migmatite, granito-gneiss, gneiss and amphibolites) described during drilling (LB) in a total of 34 boreholes, were mostly identified on the geological map (LM) as granitoids (29 boreholes) and to a lesser extent as schists (2 boreholes) and basalts (3 boreholes). As for the granitoids, of the 623 boreholes described during drilling within this group of rocks, 80 boreholes were identified on the geological map as consisting of basalt (28 boreholes), volcano-sedimentary schists (25 boreholes), gabbros (8 boreholes), leptynites (8 boreholes), rhyolites (8 boreholes) and andesites (3 boreholes).

The cause of these discrepancies between the two lithological datasets may stem from inadequate during inherenbiases within the geological map itself, hence the proposal for a methodology to correct and validate the lithological data for the study area.

#### 3.1.5 Determining the final lithology of water boreholes

The discrepancies observed between the two sets of lithological data led us to develop a methodology for correcting and validating the lithological data by integrating boreholes descriptions, field observations and boreholes silica content.

##### 3.1.5.1 Validation using field data

**Fig. 5:**
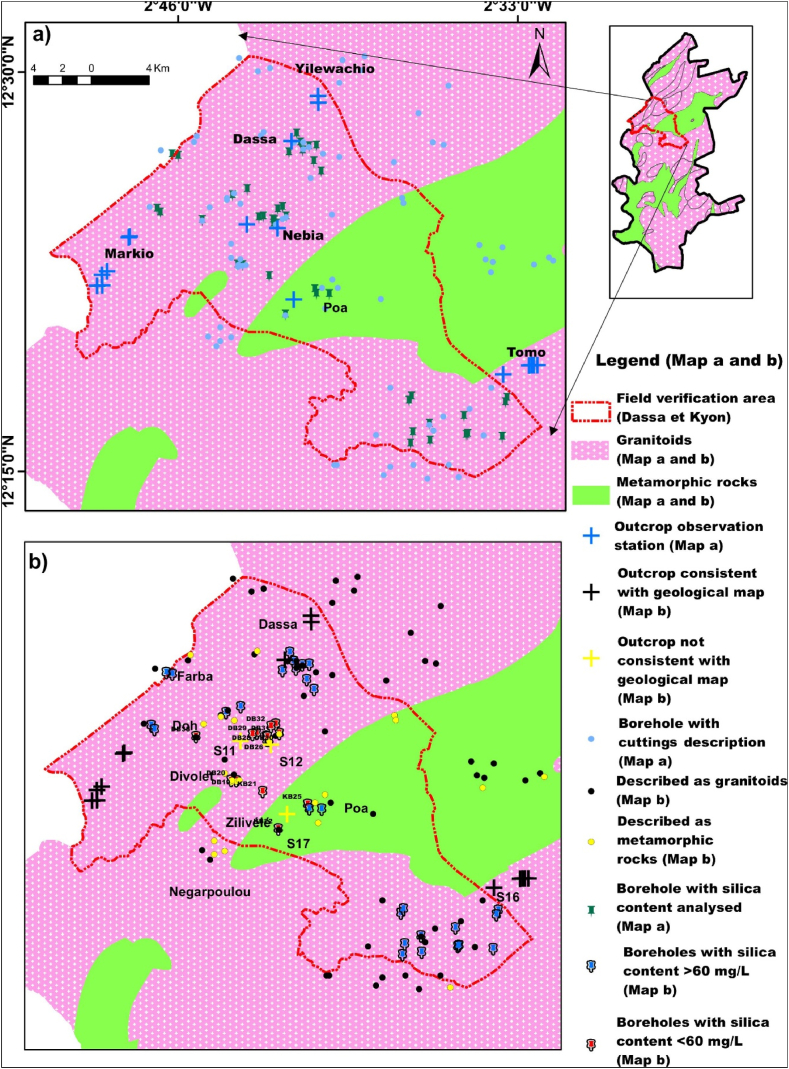
Location of outcrops, boreholes, and boreholes sampled for groundwater physico-chemical analyses on the 1:200,000 geological map of Dassa and Kyon from Ref. [13].

**Fig. 6:**
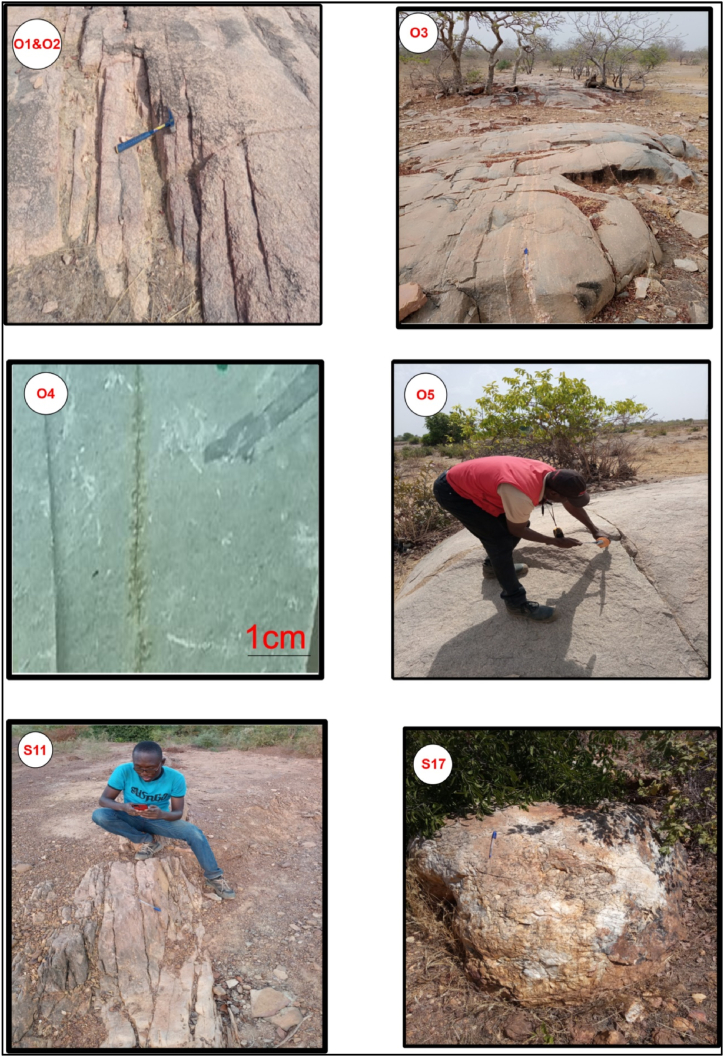
Main lithologies observed in the study area: O1, O2, O3 and O5: granites; O4 meta-basalt; S11 weathered layer of meta-basalt; S17 quartz vein (the pen and the hammer or the graphical scale give the scale).

The study area is characterised by an important lateritic cover, which means that outcrops are relatively rare. Nevertheless, fieldwork enabled us to observe 16 outcrop areas and to take 5 samples for laboratory work (Fig. 5a). Field observations and thin sections analyses show that the 14 rocks observed are granitoids and only one corresponds to a metavolcanic rock. Granitoids are leucocratic rocks with a pinkish background and a fairly coarse-grained texture. Macroscopic observations (Fig. 6-O1; O2; O3 and O5) reveals that they are composed of biotite, plagioclase and quartz. Among the granitoids, sample O3 (X = 521898 m and Y = 1370376 m) shows the beginnings of orthogneissification as it is located on a shear zone. The metavolcanic rock (Fig. 6-O4) is greenish with a microlithic texture.

**Fig. 7:**
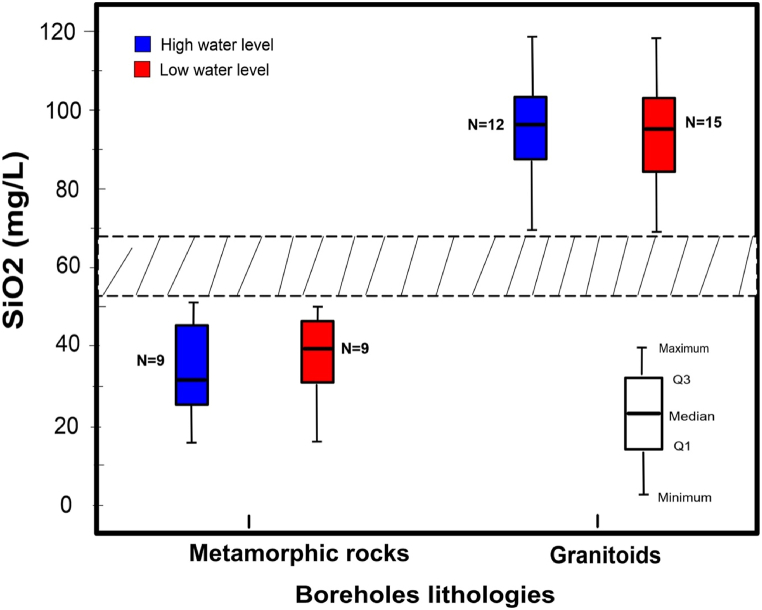
Seasonal variation in silica content at high and low water levels in boreholes for which the lithology was verified in the field. A band of silica uncertainty ranging from 52 to 72 mg/L separates the two groups of rocks.

**Fig. 8:**
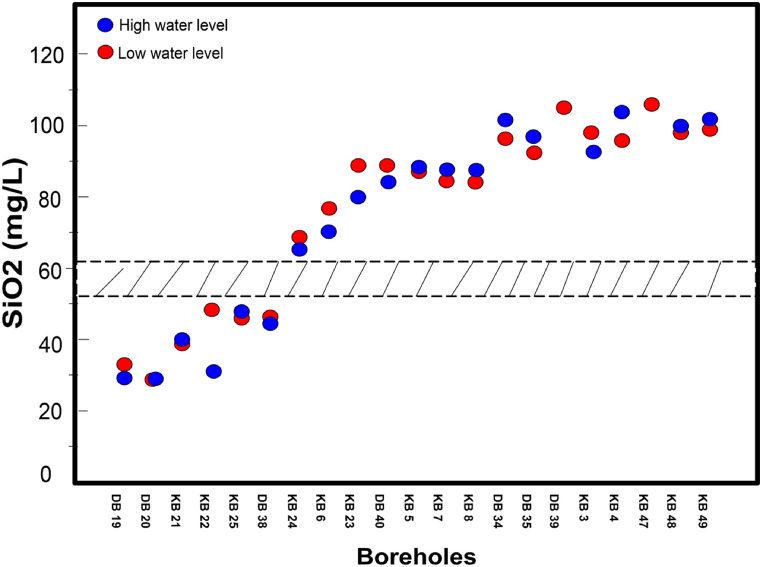
Seasonal variation in silica content at high and low water levels in boreholes where the lithology does not outcrop the ground. Then, from all these informations, it appears that the silica content threshold to distinguish metamorphic rocks from granitoids is about between 52 and 62 mg/L.

**Fig. 9:**
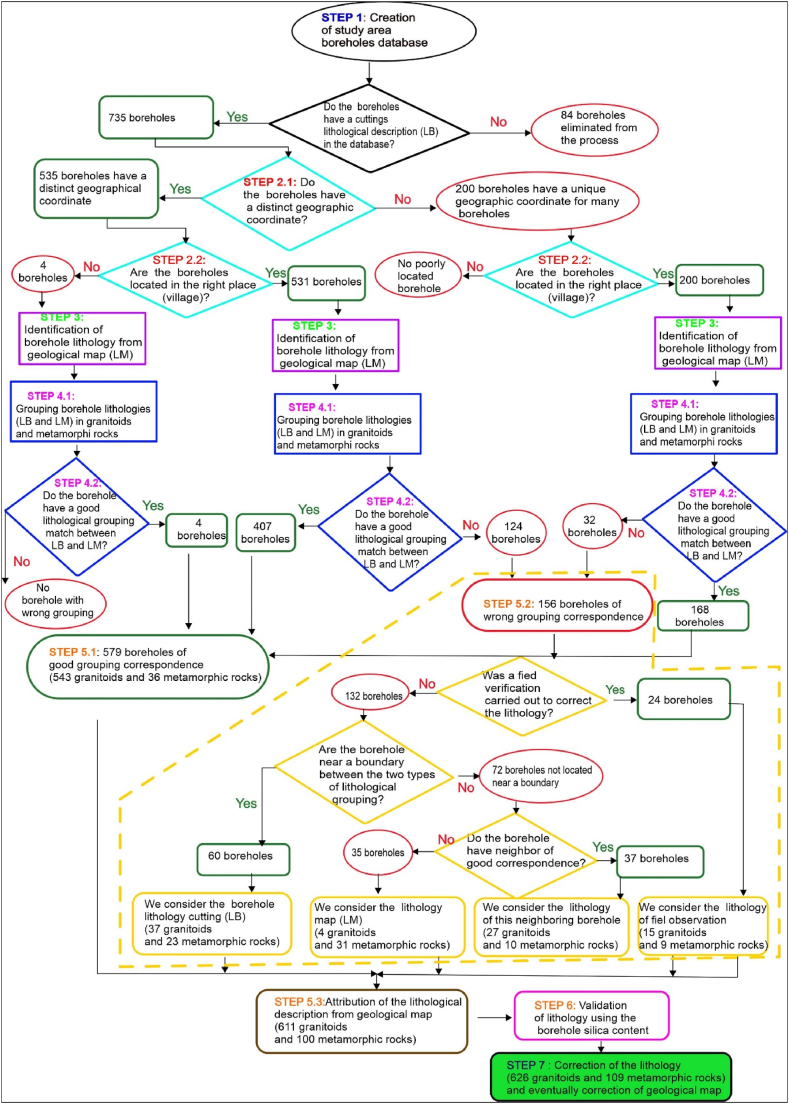
Detailed results of the application of the methodology for correcting the lithology and geological map of the Sanguie province.

**Fig. 10:**
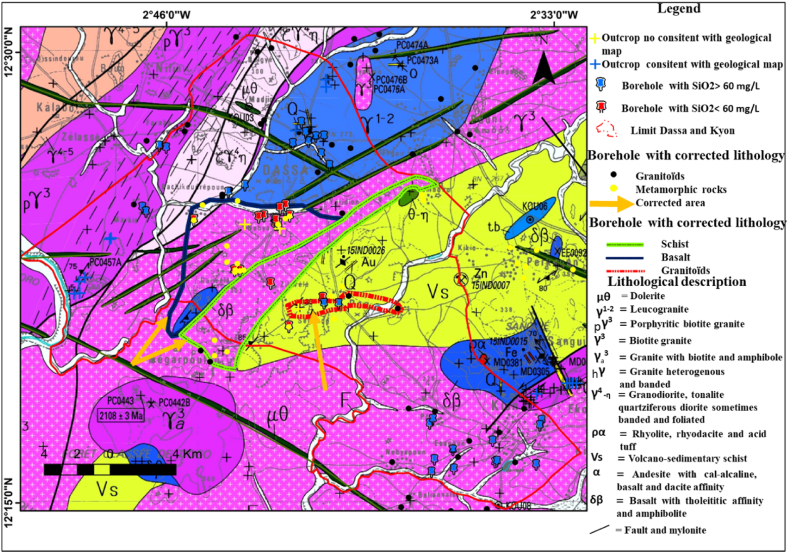
1:200,000 geological map of the communes of Dassa and Kyon (extract from Ref. [13]) with new lithological boundaries proposed.

**Fig. 11:**
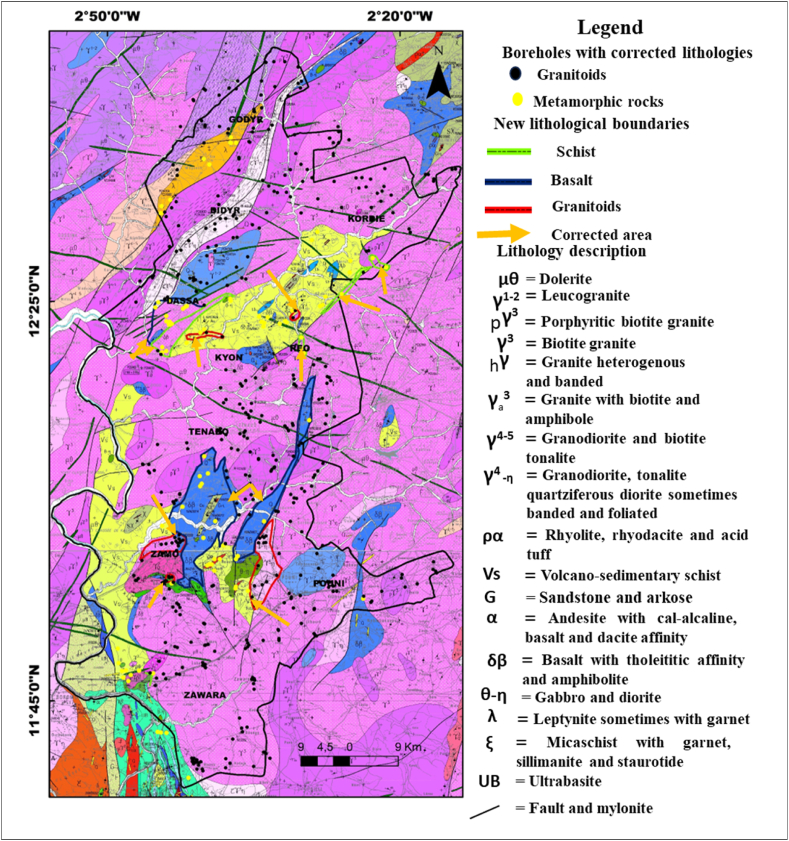
1:200,000 geological map of Sanguié province (after [13,14]) with new lithological boundaries proposed.

The lithologies described at our observation and sampling points are consistent in many cases with the data on the geological map (Fig. 5b). However, discrepancies were noted at a few locations:

- Station S12 (X = 532231 m; Y = 1371023 m), where the sample is a metabasalt, whereas the geological map indicated a granitoid environment (Fig. 5). The distance between this station and the limit of the band of volcano-sedimentary rock is approximately 3.2 km.
- station S11 (X = 530105 m; Y = 1371284 m) located approximately 4.4 km from the limit of the band of volcano-sedimentary rocks, where the outcrop, although highly weathered and purplish in colour (Fig. 6, Fig. 7, Fig. 8, Fig. 9, Fig. 10, Fig. 11), seems to have more of an affinity with the volcano-sedimentary rocks.
- Station S17 (X = 533358 m; Y = 1366095 m), where quartz veins are often metric in size, whereas the geological map indicated a volcano-sedimentary environment (Fig. 6-S17).

##### 3.1.5.2 Validation by hydrochemical approach

Chemical analyses were performed on groundwater samples from most boreholes in the study area. It turns out that only silica enables to distinguish between the two types of lithologies (granitoids and metamorphic rocks).

Fig. 7 illustrates the seasonal variation in silica content accross 24 boreholes whose geology has been verified and validated on the basis of field observations and which tap either metavolcanic rocks or granitoids. This figure shows a fairly clear distinction between the two types of aquifer lithology tapped by these boreholes in the study area. The first group, represented by the metavolcanic rocks, is characterised by low silica content ranging from 15.7 to 51.3 mg/L in high water level and from 16.1 to 50.3 mg/L in low water level. The silica content of the second group, corresponding to granitoids, is higher and varies in high water level from 73.0 to 117.7 mg/L and in low water level from 72.1 to 118.2 mg/L. A band of silica uncertainty ranging from 52 to 72 mg/L separates the two groups of rocks. It should also be noted that these concentrations, which correspond to the two main seasons, show a quite good stability over time.

Whatever the season (high or low water level), from these data, silica content is below 55 mg/L for metamorphic rocks and above 60 mg/L for granitoids. The silica content of the water thus makes it possible to distinguish the lithology of the aquifer tapped by the boreholes according to whether it is a metamorphic rock (specifically metavolcanics rock) or a granitoid. It should also be noted that all of these 24 boreholes are identified on the geological map as tapping only granitoids. Consequently, our process will enable to correct the geological map.

Fig. 8 illustrates the seasonal variation of silica content for the 21 boreholes where the lithology could not be verified in the field due to the absence of nearby outcrops. Again, there is a clear discrimination in silica content, allowing these boreholes to be categorized into two distinct silica content group. The first group consists of 6 boreholes (DB19, DB20, DB38, KB21, KB22 and KB25) with silica contents ranging from 28 to 47 mg/L at high water level and from 29 to 48 mg/L at low water level. According to Fig. 7, the silica content of these boreholes suggests they belong to metamorphic rocks, which calls into question the rock lithology from the geological map. Notably, except for boreholes KB22 and KB25, identified on the geological map as schists, the 4 others are identified as granitoids. The second group consists of 15 boreholes with silica levels ranging from 65 to 104 mg/L at high water level and from 68 to 106 mg/L at low water level, corresponding to the silica content found in boreholes tapping granitoids, as shown in Fig. 7. With the exception of boreholes KB23 and KB24 located in the village of Poa, which are situated on metamorphic rocks (volcano-sedimentary schist) on the geological map, all the boreholes in this group are in locations where there is consistency between the geological map's descriptions, the cuttings descriptions from neighbouring boreholes, and the boreholes silica content. The silica content of boreholes KB23 and KB24, where the geological map only mentions the presence of metamorphic rocks, clearly confirms that these boreholes actually intersect granitoids. In the village of Poa, 2 boreholes located not far from KB23 and KB24 are described from cuttings as tapping granitoids (Fig. 5b). This confirms the information gained from the silica content.

### 3.2 Proposal for an updated geological map of the Sanguié province

The methodology outlined for correcting and validating the lithological data from the boreholes, as well as for updating the geological map, as detailed in Fig. 9. This process successfully validated or corrected the lithological description of the 735 boreholes from our database. Finally, 627 of these boreholes are located in granitoids and 108 in metamorphic rocks.

The implementation of this new mapping methodology in the Sanguié province was followed by its verification in the communes of Dassa and Kyon. In addition to using field data as a reference, groundwater silica content was employed to determine and correct the lithology of the boreholes. These new lithological data also facilitated the correction of lithological contacts delineations proposed by Refs. [13,14] for the communes of Dassa and Kyon and the Sanguié province respectively. A geological map is a 2D projection of the earth's surface, and the borehole is a 1D depth datum. The combination of these two pieces of information for the conceptual delineation of new boundaries was achieved by considering uniform at the earth's surface, the lithology captured by the borehole. Boreholes in this area, like those in the basement zones, are very shallow (generally less than 100 m) and generally capture a single lithology [12]. The weathered layer and underlying lithology can be considered uniform on the surface and drawn as a single lithology for boreholes with the same descriptions. The proposed new boundaries were drawn up taking into account only the 531 well-located boreholes. Corrections are indicated with dashed lines in green, red and blue for volcano-sedimentary schists, granitoids and basalts respectively (Fig. 10, Fig. 11). In addition to the communes of Dassa and Kyon, these corrections are also proposed in the communes of Kordie, Réo, Zamo and Pouni. In these communes, the proposed corrections to the map plots, taking the lithology described from the borehole cuttings as the definitive lithology, compared with that on the map, when the borehole is located near a boundary between two lithologies on the geological, showed that all boreholes in this situation were located less than 1 km from the initial geological map plots. This validates the relevance of this criterion for map correction in the absence of field work or outcrops.

## 4 Discussion

In this study, we demonstrate the value of lithological data from water boreholes in improving the geological map of the Sanguié province. More than 84 % of the dataset used is made up of such lithological data from water boreholes drilled during village water supply campaigns in the Sanguié province. Most of these boreholes were drilled before 2003, the date of the geological map. 78 % of them corresponded very well with the description on the 2003 geological map. This shows that these descriptions are fairly reliable and in line with the reality on the ground, and that the data can be used to support conventional mapping tools to improve the production of geological maps. These data also show a very good coverage of the study area and have the added advantage of being often the only means of accessing the lithology in the absence of outcrops in the field. This scarcity of outcrops is almost a general situation throughout Burkina Faso. In fact, in the western region of Burkina Faso [10], estimate the outcrop rate at around 6 %. The Koudougou sheet is no exception to this situation. It is characterised by the presence of numerous areas with extensive lateritic cover. Very little information was provided about these areas when the 2003 map was produced, and only 10 boreholes with core recovery were drilled in these areas for the production of the map [9]. Still according to these authors, the geological mapping in these zones with a thick weathering cover was mainly done on the basis of the interpretation of airborne geophysics. These reasons show that taking into account the lithological data from water boreholes is an added value for improving the geological map. Maps that take into account several data sources are more reliable, especially when they incorporate details from geological mapping areas that are richer in data [45].These maps are also more accurate when they include drilling and/or geophysical data in their production process [8,46].

The hydrochemical approach, using silica content, enabled the lithology of the geological units in the study area to be clearly distinguished. The two main lithological units are chemically distinct in terms of their silica content. Granitic facies, which are higher in silica clearly contrast with metamorphic rock facies, which are poorer in silica. The silica content in groundwater proved to be a good discriminant between these two lithological groups. However, the method has its limitations when one considers that metamorphic rocks include a number of rock types that were not sampled in our study area. For example, metarhyolite and leptynite formations may exhibit a similar silica content that granitoïds as these have the same composition as the granitoids. However, the latter are only sparsely represented among the basement rocks that generally outcrop in the West African craton [47,48], and particularly in Burkina Faso.

This correlation between lithology and silica content was previously established by Ref. [39] for the aquifer formations of the Sourou watershed in the Mouhoun region. The chemical compositions of the rocks making up these different lithologies have already been characterised by Ref. [29], who showed that the metamorphic rocks are mainly basic to intermediate. The silica content of the groundwater reflects the chemical composition of the two types of aquifer formations. Granitoids generally exhibit a silica content higher than 60 % [[49], [50], [51], [52], [53]], while metavolcanic rocks may display slightly lower silica levels [29,[54], [55], [56], [57], [58]].This robust correlation between water silica content and lithology is attributed to water-rock interaction processes, which are one of the main factors guiding groundwater chemistry [36,40,59,60]. The hydrochemical quality of groundwater is mainly due to the geological nature of the rock that forms its reservoir. The duration of the interaction process between water and rock contributes to the dissolution of the rock's chemical elements and consequently to the mineralization of the water circulating in the rock. In terms of silica content, supersaturated rocks such as granitoids and their volcanic equivalents, will display similar silica content in borehole water in which they form aquifers, as undersaturated and intermediate rocks (basalts, andesites, peridotites …).

Discrimination of aquifer lithology of the tapped by boreholes based on silica content has also confirmed the validity of this new mapping approach based on the lithology of the water boreholes, and has significantly reduced the biases resulting from the compilation of several data sources. This data validation stage is very important when building a multi-source database [61]. [8] have shown that there is a good correlation between maps created from water borehole data and geological atlas maps. Thus, the density of drilling data is an important means of increasing the resolution of these atlases. Although drilling data can come from a variety of sources, it is complementary data that greatly improves the quality of the maps produced [62].

This geological mapping methodology presented in this paper is fairly simplistic, and offers the advantage of refining the contours of the lithological contacts on the geological map while remaining in the same order of scale at which the map was produced. This approach to updating the geological map using water borehole lithology further demonstrates that water borehole data can be of very high interest to geological mapping. However, the applicability of our methodology to other types of geological formations, such as sedimentary rocks for instance, hosting so-called continuous aquifers, would not be very obvious for two mains reasons:

- Firstly, sedimentary layers may be very thin. Our method should then be adapted as only the upper layer should be represented on the geological map;
- secondly, continuous aquifers may allow the flow of groundwater from far away. Consequently, the groundwater hydrochemistry may surely not be a pertinent parameter such as in crystalline and metamorphic aquifers. Moreover, the aquifers may not be the first layer at the ground surface, thus the one to be represented on the geological map. However, the applicability of our approach to sedimentary formations could be an interesting research prospect.

## 5 Conclusions

The Sanguié province, like the rest of Burkina Faso, is characterized by a geology composed mainly of granitoids, bordered by more or less narrow belts of metamorphic rocks (metavolcanites and metavolcano-sediments). Due to the extensive regolith cover, these rocks are rarely exposed at the surface and have largely been mapped using airborne imagery interpretations.

The present study utilizes lithological descriptions obtained during the drilling of boreholes as part of village hydraulics campaigns, hitherto little used in geological mapping in Burkina Faso, to improve the geological map of the Sanguié province. Additionally, hydrochemical analysis data, particularly silica content of boreholes, were employed as a good discriminant of the main lithologies. Fieldwork was also necessary to find a third element of appreciation and validate the proposed approach.

At the end of this study, we note that cuttings data from water wells, completed with silica content in groundwater, constitute a fairly reliable source and database for geological mapping. These data are of vital interest and can be used to complement conventional mapping techniques in the production of geological maps of outcrop-poor basement areas across the country. The methodology proposed in this work for updating the geological map of the Sanguié province can thus be extended to other basement provinces in Burkina Faso and elsewhere in the world, where sufficient borehole lithology is available.

As another perspective, water borehole data can also be used to provide regolith cover thickness maps (both the saprolite, and the underlying fractured layer), as [19] did.

## CRediT authorship contribution statement

**Cheik Abba Cissé Ouangaré:** Writing – review & editing, Writing – original draft, Methodology, Funding acquisition. **Séta Naba:** Writing – review & editing. **Patrick Lachassagne:** Writing – review & editing.

## Data availability

The data associated with this study has not been deposited in a publicly accessible repository. They can be made available on request.

## Funding

This paper is the result of the thesis work of Mr OUANGARE, whose work (fieldwork, chemical analysis) and research stays at the Unité Mixte de Recherche HydroSciences Montpellier (UMR-HSM) were funded respectively by the Drinking Water Supply and Sanitation Program (10.13039/100013788PAEA) of the Ministry in charge of water in Burkina Faso and the 10.13039/501100004493International Atomic Energy Agency (10.13039/501100004493IAEA).

## Declaration of competing interest

The authors declare that they have no known competing financial interests or personal relationships that could have appeared to influence the work reported in this paper.
